# Stereotactic body radiotherapy versus conventional/moderate fractionated radiation therapy with androgen deprivation therapy for unfavorable risk prostate cancer

**DOI:** 10.1186/s13014-020-01658-5

**Published:** 2020-09-15

**Authors:** Sagar A. Patel, Jeffrey M. Switchenko, Ben Fischer-Valuck, Chao Zhang, Brent S. Rose, Ronald C. Chen, Ashesh B. Jani, Trevor J. Royce

**Affiliations:** 1 Department of Radiation Oncology, Winship Cancer Institute, Emory University, Atlanta, GA USA; 2 Department of Biostatistics and Bioinformatics, Emory University, Atlanta, GA USA; 3 Department of Radiation Medicine and Applied Sciences, University of California San Diego, San Diego, CA USA; 4 Department of Radiation Oncology, University of Kansas, Kansas City, KS USA; 5 Department of Radiation Oncology, University of North Carolina, Chapel Hill, NC USA

**Keywords:** Ultrahypofractionation, Prostate cancer, High risk

## Abstract

**Background:**

Ultrahypofractionation using stereotactic body radiotherapy (SBRT) is an increasingly utilized technique for men with prostate cancer (PC). The comparative efficacy of SBRT plus androgen deprivation therapy (ADT) compared to fractionated radiotherapy (EBRT) plus ADT in higher-risk prostate cancer is unknown.

**Methods:**

Men > 40 years old with localized PC treated with external beam radiation and concomitant ADT for curative intent between 2004 and 2016 were analyzed from the National Cancer Database. Patients who lacked ADT or risk stratification data were excluded. 558 men treated with SBRT versus 40,797 men treated with conventional or moderately hypofractionated EBRT were included. Patients were stratified by unfavorable intermediate (UIR) and high (HR) risk using NCCN criteria. Kaplan Meier and Cox proportional hazards were used to compare overall survival (OS) between RT modality, adjusting for age, race, and comorbidity index.

**Results:**

With a median follow up of 74 months, there was no difference in estimated 6-year OS between men treated with SBRT versus EBRT regardless of risk group. On multivariable analysis, there was no difference in risk of death for men treated with SBRT compared to EBRT (UIR: adjusted HR 1.09, 95% CI 0.68–1.74, *p* = .72; HR: adjusted HR 0.93, 95% CI 0.76–1.14, *p* = .51). On sensitivity analyses, when confining the cohort to men treated with NCCN-preferred dose fractionations, with no comorbidities, or < 65 years old, there remained no survival difference between treatment groups for both UIR and HR.

**Conclusion:**

Within study limitations, we found no difference in survival between SBRT+ADT and standard of care EBRT+ADT for UIR or HR PC. These results support recent NCCN guideline updates, which include SBRT as a non-preferred option for higher risk men. Prospective validation would further strengthen the evidence basis behind these recommendations.

## Introduction

Hypofractionated radiation therapy for prostate cancer is an appealing and increasingly adopted approach that has advantages from a radiobiologic, cost, and patient convenience standpoint [1–4]. Non-inferiority phase 3 randomized trials have confirmed the safety and efficacy of moderate hypofractionation (2.5–3 Gy per fraction) compared to conventional fractionation (1.8–2 Gy per fraction) [5–7]. Furthermore, one randomized trial showed superior biochemical control with moderate hypofractionation compared to conventional fractionation [8]. Moderate hypofractionation has now been accepted as a standard-of-care across all risk groups and a preferred regimen in ASTRO and NCCN Guidelines [9, 10].

More recent randomized trials have shown that ultrahypofractionated radiation (≤7 fractions, ≥5 Gy per fraction), or stereotactic body radiation (SBRT) when delivered in ≤5 fractions with image/stereotactic guidance, is non-inferior to conventional fractionation for tumor control and toxicity, and to moderate hypofractionation for toxicity [11, 12]. There is increasing interest in ultrahypofractionated radiation therapy for prostate cancer to further optimize patient convenience and cost effectiveness [13]. The ASTRO/ASCO/AUA societal guidelines do not currently recommend routine use of ultrahypofractionated radiation therapy for men with unfavorable risk prostate cancer, with a conditional recommendation *against* its use in men with high risk disease [9]. Since publication of those guidelines, however, the HYPO-RT-PC randomized trial [11] showed non-inferiority of ultrahypofractionation in a cohort of intermediate and high risk men. However, androgen deprivation therapy (ADT), which is standard in the United States in these men, was not permitted in that trial. Furthermore, only 11% of enrolled men on that trial had NCCN-defined high risk disease. How ultrahypofractionation plus ADT compares with conventional/moderate fractionation plus ADT in men with higher risk prostate cancer remains unknown. Herein, we examine outcomes between these two approaches in men with UIR and HR prostate cancer who receive concomitant ADT. We hypothesize that ultrahypofractionation has similar outcomes as conventional/moderate fractionation for these men.

## Methods

Men > 40 years with localized prostate cancer treated with external radiation and ADT with curative intent between 2004 and 2016 were analyzed from the National Cancer Database. Patients who received brachytherapy, surgery, chemotherapy, or immunotherapy were excluded. Patients missing ADT or risk stratification data were excluded. Those that received ADT > 180 days before or after the start of radiation were excluded. Ultrahypofractionation (SBRT) was defined as 5 fractions of ≥5 Gy per fraction and conventional/moderate fractionation (EBRT) as ≤3 Gy per fraction and total dose ≥60 Gy. Patients were stratified by risk using NCCN criteria: unfavorable intermediate (UIR) and high (HIR).^1^ ANOVA and chi square test was used to compare patient/demographic characteristics. Cochran-Armitage test for trend was used to evaluate utilization of SBRT in this cohort between 2004 and 2016. Kaplan Meier and multivariable Cox proportional hazards were used to compare overall survival (OS) between those who received EBRT versus SBRT, accounting for age, race, comorbidity index, and year of diagnosis. All analyses were computed using SAS 9.4 (SAS Institute Inc., Cary, NC). Tests were 2-sided with a 0.05 level of significance. This study received IRB exemption.

## Results

Forty-one thousand three hundred fifty-five men were eligible for this analysis: 40,797 treated with EBRT and 558 treated with SBRT (Table 1). Although SBRT has been minimally utilized in UIR and HIR prostate cancer between 2004 and 2016, there has been a significant rise in its use over this time (*p* for trend <.001). There was an uptick in use of SBRT in UIR men after 2011–2012 (Supplemental Figure). A larger proportion of men in the SBRT cohort were Black, treated at an academic center, had median household incomes ≥$46,000, were treated in the Northeast and West United States, lived > 50 miles away from treatment facility, and resided in metro/urban over rural areas (Table 1).

**Table 1:** Patient clinical and demographic characteristics

Covariate	Statistics	Level	Radiation group		Parametric ***P***-value^**a**^
			SBRT ***N*** = 558	EBRT ***N*** = 40,797	
Race	N (Col %)	White	415 (75.73)	32,076 (79.47)	0.07
	N (Col %)	Black	114 (20.8)	6923 (17.15)	
	N (Col %)	Other	19 (3.47)	1363 (3.38)	
Facility Type	N (Col %)	Non-academic program	237 (42.47)	28,382 (69.57)	**<.001**
	N (Col %)	Academic program	321 (57.53)	12,415 (30.43)	
Median Income Quartiles	N (Col %)	< $30,000	76 (14.07)	5527 (13.96)	**<.001**
	N (Col %)	$30,000 - $34,999	60 (11.11)	7315 (18.47)	
	N (Col %)	$35,000 - $45,999	130 (24.07)	11,117 (28.07)	
	N (Col %)	> = $46,000	274 (50.74)	15,643 (39.5)	
Year of diagnosis	N (Col %)	2004–06	92 (16.49)	8811 (21.6)	**<.001^**
	N (Col %)	2007–09	109 (19.53)	9121 (22.36)	
	N (Col %)	2010–12	127 (22.76)	10,852 (26.6)	
	N (Col %)	2013–15	230 (41.22)	12,013 (29.45)	
Insurance status	N (Col %)	Not Insured	12 (2.2)	744 (1.85)	0.787
	N (Col %)	Private	146 (26.79)	10,563 (26.25)	
	N (Col %)	Government insurance	387 (71.01)	28,931 (71.9)	
Facility Location	N (Col %)	Northeast	188 (33.69)	10,369 (25.42)	**<.001**
	N (Col %)	South	155 (27.78)	13,291 (32.58)	
	N (Col %)	Midwest	112 (20.07)	10,751 (26.35)	
	N (Col %)	West	103 (18.46)	6386 (15.65)	
Risk group	N (Col %)	Unfavorable intermediate	130 (23.3)	5094 (12.49)	**<.001**
	N (Col %)	High	428 (76.7)	35,703 (87.51)	
Distance to facility	N (Col %)	0–10 miles	250 (44.88)	22,460 (55.25)	**<.001**
	N (Col %)	10–50 miles	212 (38.06)	15,550 (38.25)	
	N (Col %)	50+ miles	95 (17.06)	2641 (6.5)	
Charlson comorbidity score	N (Col %)	0	472 (84.59)	34,616 (84.85)	0.197
	N (Col %)	1	74 (13.26)	4825 (11.83)	
	N (Col %)	2+	12 (2.15)	1356 (3.32)	
Urban/Rural Location	N (Col %)	Metro	439 (82.06)	31,912 (80.12)	**0.022**
	N (Col %)	Urban	92 (17.2)	6854 (17.21)	
	N (Col %)	Rural	4 (0.75)	1064 (2.67)	
Age at Diagnosis	N		558	40,797	0.980
	Mean		70.18	70.19	
	Median		71	71	
	Min		43	40	
	Max		90	90	
	Std Dev		7.95	7.91	

^a^ Parametric p-value calculated by ANOVA for numerical covariates and chi-square test for categorical covariates

^ *P*-value calculated by Cochran-Armitage test for trend

**Fig. 1:**
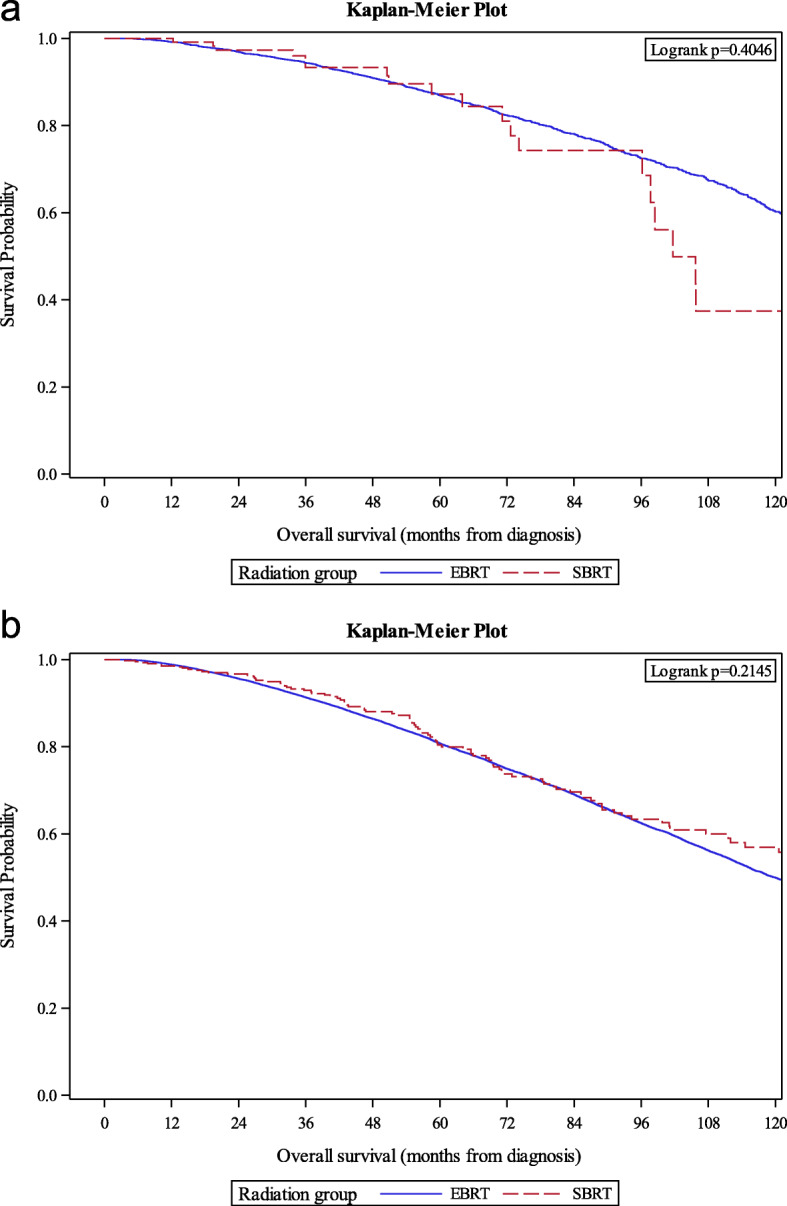
Kaplan-Meier survival estimates between EBRT and SBRT for unfavorable intermediate (**a**) and high (**b**) risk prostate cancer

The median follow up time was 74 months. There was no difference in estimated 6-year OS between men treated with SBRT versus EBRT regardless of risk group (SBRT versus EBRT, UIR: 93.3% versus 90.9%, log-rank *p* = .40, Fig. 1a; HIR: 80.8% versus 80.4%, log-rank *p* = .21, Fig. 1b). On multivariable analysis, accounting for age, race, and comorbidity, there was no difference in mortality for men treated with SBRT compared to EBRT (UIR: adjusted HR 1.09, 95% CI 0.68–1.74, *p* = .72; HIR: adjusted HR 0.93, 95% CI 0.76–1.14, *p* = .51). On sensitivity analyses, when 1) excluding SBRT < 7 Gy per fraction, 2) excluding EBRT < 74 Gy if < 2 Gy per fraction, 3) limiting the cohort to < 65 years of age, and 4) limiting the cohort to those with no medical comorbidities, no significant difference in OS was found between SBRT and EBRT (Table 2).

**Table 2:** Multivariable overall survival (OS) analysis between SBRT+ADT versus EBRT+ADT (referent) in overall cohort and select subgroups for unfavorable intermediate (a) and high (b) risk prostate cancer

	HR	95% CI	***p***
**a. Unfavorable Intermediate Risk**			
Overall	1.09	0.68–1.74	.72
*Subgroup Analyses*			
Excluding any SBRT < 7 Gy per fraction	1.15	0.72–1.83	.57
Excluding any EBRT < 2 Gy per fraction *and* < 74 Gy total dose	1.24	0.79–1.96	.35
Excluding Charlson Deyo ≥1	1.29	0.89–1.34	.33
Excluding age ≥ 65	1.13	0.86–1.36	.60
**b. High Risk**			
Overall	0.93	0.76–1.14	.51
*Subgroup Analyses*			
Excluding SBRT if < 7 Gy per fraction	0.92	0.74–1.14	.44
Excluding EBRT if < 2 Gy per fraction *and* < 74 Gy total dose	0.90	0.74–1.10	.30
Excluding Charlson Deyo ≥1	0.83	0.66–1.03	.10
Excluding age ≥ 65	0.72	0.44–1.17	.18

## Discussion

We found no difference in survival between SBRT+ADT and standard of care EBRT+ADT for UIR or HIR PC. ASTRO/ASCO/AUA consensus guidelines, though outdated, do not recommend routine use of SBRT for higher risk prostate cancer. Conversely, recent NCCN guidelines provide support of SBRT for UIR and HIR patients, particularly when more protracted courses may provide social or medical hardship [10]. The NCCN note that moderate fractionation is the preferred external beam radiation therapy regimen for all risk categories. Our results reinforce the NCCN’s recent decision to endorse SBRT as an option for men with higher risk prostate cancer and may motivate ASTRO to reconsider their guidelines.

More widespread SBRT use in these patients may be appropriate after publication of the HYPO-RT-PC trial that showed non-inferiority of ultrahypofractionation compared to conventional fractionation after a median follow up of 5 years. ADT use, which is standard for these patients in the United States, was not permitted in that study. Prospective data regarding SBRT with concomitant ADT is lacking; data showing favorable outcomes with SBRT, though with inconsistent ADT use, for higher risk prostate cancer is largely retrospective [14]. Our study corroborates institutional results regarding comparable disease control and survival with SBRT compared to conventional/moderate hypofractionation.

There are several potential advantages of SBRT. For one, the alpha-beta ratio of prostate cancer may potentially be lower than for late normal tissue reactions [15]. If true, ultrahypofractionation could increase the therapeutic ratio and thereby offer more efficacious local therapy. Second, despite the use of complex immobilization, on-board imaging, and physics resources, SBRT reduces overall costs to payers and patients, with up to half the cost per allowable Medicare fee schedules, largely due to its abbreviated treatment schedule [16, 17]. In an era of rising healthcare costs, as well as anticipated Alternative Payment Models with bundled fee schedules, providers will be incentivized to utilize the most cost-effective options. Finally, with reduced treatment visits, SBRT provides a more convenient treatment option for patients compared to protracted fractionation schemes.

Based on recently available level one evidence published in 2019, specifically PACE-B and HYPO-RT-PC, SBRT should be more widely accepted as an appropriate regimen for PC in patients eligible for prostate +/− seminal vesicle treatment alone, regardless of risk group. This is relevant in an era of optimal locoregional imaging, namely MRI, which can help rule out high risk features that may otherwise support larger treatment margins and/or pelvic nodal irradiation. Even for patients who may require pelvic nodal treatment, however, the SATURN trial has shown safety and promising efficacy of elective nodal irradiation utilizing ultrahypofractionation [18]. For PC there is a radiobiologic advantage of ultrahypofractionation over protracted courses utilizing smaller doses per treatment. Now, there is prospective basis for its use.

One concern that may limit utilization of SBRT for localized prostate cancer is toxicity. The HYPO-RT-PC trial [11] showed higher patient-reported urinary and bowel toxicity with ultrahypofractionation, with higher urinary toxicity extending to 1 year after completion of treatment; late toxicity, however, was similar between ultrahypofractionation and conventional fractionation. PACE-B [12], on the other hand, showed non-inferior toxicity within the first 12 weeks after treatment between SBRT and conventional/moderate fractionation for favorable risk prostate cancer. The discrepancy in acute toxicity between these two studies may be due to the time frame of each trial accrual, with patients enrolled on HYPO-RT-PC treated between 2005 and 2015 and those enrolled on PACE-B treated between 2012 and 2018. Approximately 80% of men on HYPO-RT-PC received 3-dimensional conformal RT; advancements in treatment delivery, including intensity modulation, between these two eras may explain the discrepancy in acute toxicity findings [19]. Furthermore, recent multi-institutional analysis of prospectively-collected data of over 2000 men treated with SBRT showed very low rates of grade 3 genitourinary and gastrointestinal toxicity after 7 years of follow up [20]. Integration of rectal spacer or balloon, as allowed in the NRG GU005 phase 3 trial, may lower toxicity even further. Whether the addition of androgen deprivation therapy, postulated to function as least partly through radiosensitization [21], may increase acute/late toxicities when delivered with SBRT is unknown and remains subject for future analysis; however, based on similar toxicity seen between moderate and conventional fractionation when delivered with concomitant ADT [4–6], this likelihood is low.

This analysis has several limitations. First, given the retrospective design using a population-based database, analyses are subject to selection biases and imbalances in unmeasured variables. However, multivariate modeling was utilized to address potential confounding. Furthermore, we completed stringent sensitivity analyses confining the cohort to those treated with modern-day doses, as well as excluding older and comorbid patients, with consistent results. Second, outcome measures in the NCDB are limited to OS, so details regarding biochemical control and toxicity unavailable. While we believe OS is a primary outcome of measure in these higher risk patients to influence management decisions, several of these unavailable data are relevant in this cohort given that treatment decisions often consider patient quality of life.

## Conclusion

In conclusion, we found no difference in survival between SBRT+ADT and standard of care EBRT+ADT for UIR or HIR PC. SBRT offers a cost-effective, convenient option for prostate cancer patients in centers that are able to deliver safe therapy with precise, image-guided techniques. SBRT has wide guideline support for low and favorable intermediate risk prostate cancer. For UIR and HIR prostate cancer, however, there is historically low utilization and reserved support for SBRT use, with conditional recommendation *against* its use by ASTRO/ASCO/AUA task force. HYPO-RT-PC trial provides level one support for SBRT for unfavorable intermediate and high risk prostate cancer, but ADT was not permitted in that study. How SBRT plus ADT compares against conventional/moderate fractionated EBRT plus ADT is unknown, but our results suggest that long-term outcomes may not differ. These findings are concordant with the updated NCCN guidelines, which list SBRT as an option in men with higher risk disease.

## Supplementary information

**Additional file 1 Supplemental Figure.** Utilization of standard or moderately hypofractionated radiation (EBRT) versus ultrahypofractionated radiation (SBRT) in men with unfavorable intermediate (a) and high (b) risk prostate cancer receiving androgen deprivation therapy.

## Data Availability

Research data are available in the National Cancer Database via Common on Cancer accredited facilities.
