# Supplementary figures and images for: Stereotactic body radiotherapy versus conventional/moderate fractionated radiation therapy with androgen deprivation therapy for unfavorable risk prostate cancer
Source: Radiat Oncol. 2020 Sep 15;15:217. doi: 10.1186/s13014-020-01658-5 (PMC7493337; doi:10.1186/s13014-020-01658-5)

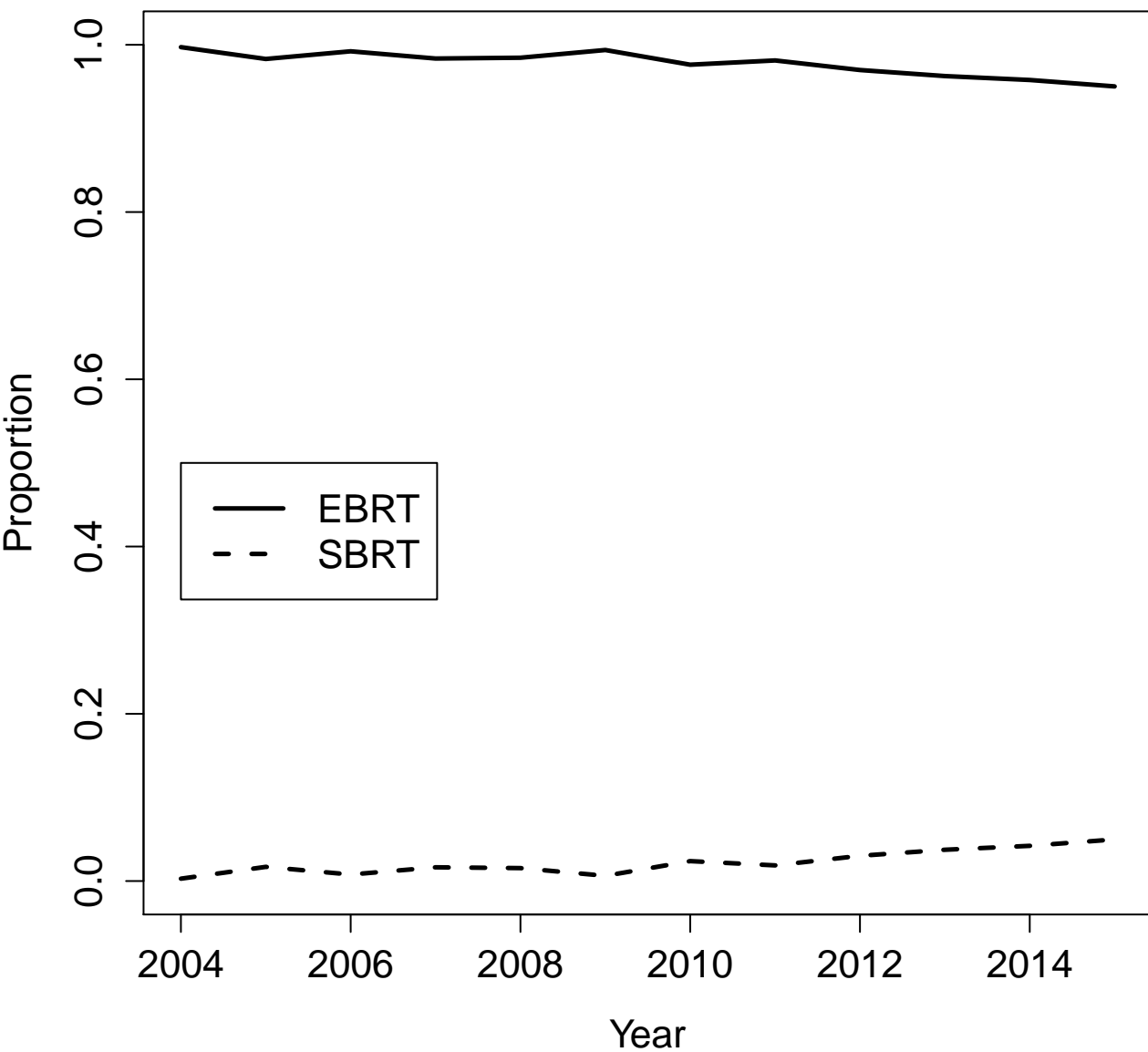

Supplement: Supplementary file 1 — Additional file 1 Supplemental Figure. Utilization of standard or moderately hypofractionated radiation (EBRT) versus ultrahypofractionated radiation (SBRT) in men with unfavorable intermediate (a) and high (b) risk prostate cancer receiving androgen deprivation therapy. [file 13014_2020_1658_MOESM1_ESM.zip › SuppFigure a.pdf]

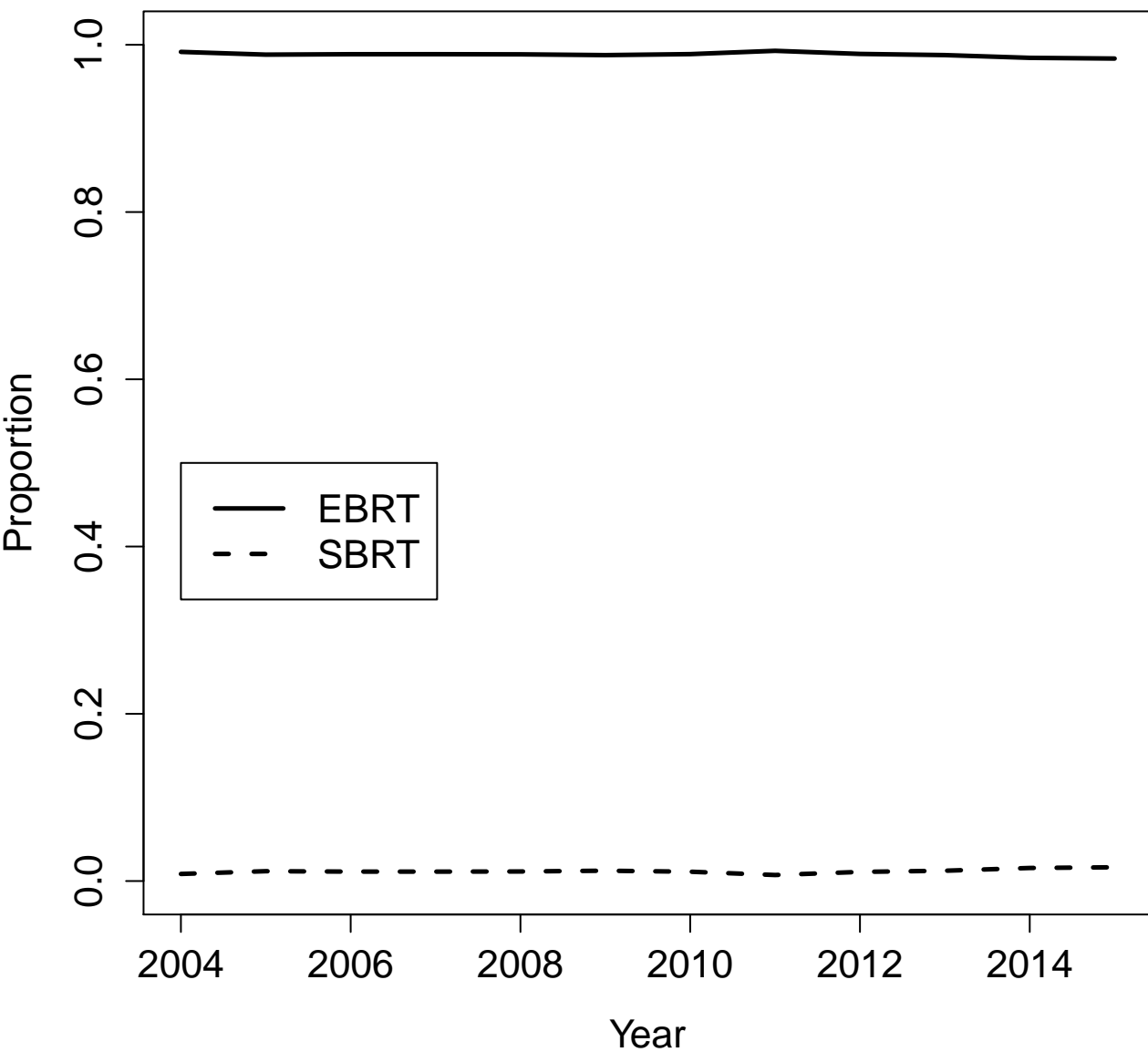

Supplement: Supplementary file 1 — Additional file 1 Supplemental Figure. Utilization of standard or moderately hypofractionated radiation (EBRT) versus ultrahypofractionated radiation (SBRT) in men with unfavorable intermediate (a) and high (b) risk prostate cancer receiving androgen deprivation therapy. [file 13014_2020_1658_MOESM1_ESM.zip › SuppFigure b.pdf]
